# Low pH Water Impairs the Tactile Sense of the Postlarvae of the Giant Freshwater Prawn *Macrobrachium rosenbergii*

**DOI:** 10.21315/tlsr2018.29.1.7

**Published:** 2018-03-02

**Authors:** Gunzo Kawamura, Teodora Bagarinao, Annita Seok Kian Yong, Siti Narasidah Noor, Leong-Seng Lim

**Affiliations:** 1 Borneo Marine Research Institute, Universiti Malaysia Sabah, 88400 Kota Kinabalu, Sabah, Malaysia; 2 Aquaculture Department, Southeast Asian Fisheries Development Center, Tigbauan, Iloilo, Philippines

**Keywords:** Mechanoreceptor, Touch Stimulus, Escape Jumping, Shadow Photography

## Abstract

The effect of low pH on the tactile sense of *Macrobrachium rosenbergii* postlarvae was determined in the laboratory by means of two behavioural assays: shelter (netting) occupancy and jumping response to touch stimuli (taps) by a glass micropipette. The postlarvae were acclimated to pH 4, pH 5, pH 6 and pH 7.5 (control) in 45 L aquaria 5–7 d before the experiments. Shelter occupancy decreased with pH and was significantly lower at pH 4 and pH 5 than at pH 6 and in the control. The jumping response instantly followed a tap 93–98% of the time in the control, pH 6 and pH 5 treatments. However, the postlarvae showed significantly lower jumping response (65%) at pH 4, indicating an impaired tactile sense. Low pH 4–5 probably degrades the chitin of the sensory setae and inhibits the surface mechanoreceptors of the prawn postlarvae.

## Abstract (ru)

Kesan pH rendah terhadap deria sentuhan pasca larva *Macrobrachium rosenbergii* telah ditentukan dalam makmal melalui dua ujian tingkah laku: penghunian pada substrat (jala) dan respon melompat terhadap sentuhan oleh mikropipet kaca. Pasca larva telah diaklimitasi kepada pH 4, pH 5, pH 6 dan pH 7.5 (kawalan) dalam akuaria 45 L selama 5–7 hari sebelum eksperimen. Bilangan pasca larva yang menghuni pada substrat berkurangan bersama pH dan adalah lebih rendah secara ketara pada pH 4 dan pH 5 berbanding pH 6 dan kawalan. Respon melompat yang segera terhadap 93–98% daripada jumlah sentuhan telah diperhatikan pada pasca larva dalam kumpulan kawalan, pH 6 and pH 5. Namun, pasca larva dalam kumpulan pH 4 menunjukkan jumlah bilangan respon melompat yang lebih rendah secara ketara (65%), menunjukkan penjejasan dalam deria sentuhan mereka. pH yang rendah (dalam pH 4–pH 5) mungkin menjejaskan kitin dalam seta deria dan menghalang permukaan reseptor mekanikal pasca larva udang.

## INTRODUCTION

Mechanoreceptors in crustaceans detect hydrodynamic signals that carry important abiotic and biotic information such as the presence and movements of prey, predators, aggressors, and potential mates (Douglass & Wilkins 1998; Herberholz & Schmitz 1998; Arnott *et al*. 1999; Prakash & Kumar 2013). The mechanoreceptors of crustaceans are cuticular hair-like projections (Garm 2004) found all over the body surface intermingled with chemoreceptors (Breithaupt & Tautz 1990). Spectrophotometry study demonstrated that the shell quality of the giant freshwater prawn *Macrobrachium rosenbergii* postlarvae and early juveniles was significantly affected by low pH especially at pH 4 (Kawamura *et al*. 2015). It is highly likely that the mechanoreceptors would be negatively affected by low pH water. Inhibition of chemosensitivity by reduced pH is well documented in crustaceans (Allison *et al*. 1992; de la Haye *et al*. 2011; Newman & Dubuque 2013), but no studies have been done on mechanoreception.

*M. rosenbergii* can be found in many freshwater resources, including lakes, ponds, rivers and estuaries in the south and southeast Asia, northern Oceania, and western Pacific islands. The berried females migrate downstream to estuaries to release eggs, and the larvae hatch as zoea. After the zoea grow and reach to the postlarval stage, they migrate into rivers and lakes (Ismael & New 2000). However, the occurrence of acid rain results in acidification of river and lake waters (Sin & Agrawal 2008), and it is now recognized as a serious threat to aquatic ecosystems (Alston & Sampaio 2000). In fact, the low pond water pH due to acid rain was reported to affect the aquaculture of *M. rosenbergii* in Taiwan (Chen & Chen 2003).

In Malaysia, acidification of river and lake waters also has been reported in several places, including in Chini Lake, Pahang (Shuhaimi-Othman *et al*. 2008), the remote Danum Valley in Sabah (Sumari *et al*. 2009), and the Wildlife Sanctuary Sibuti Mangrove Forest in Miri, Sarawak (Gandaseca *et al*. 2011). Although the aquaculture of *M. rosenbergii* now is depending on the hatchery-produced postlarvae and juveniles, the wild populations of *M. rosenbergii* can be affected by the acidification (Alston & Sampaio 2000).

This study assessed the effects of low pH rearing water (at pH 6, pH 5, and pH 4; against the control pH at 7.5) on the tactile behaviour of *M. rosenbergii* postlarvae in terms of shelter occupancy and escape jumping in response to touch stimuli. The tactile sense discriminates stimuli impinging on the mechanoreceptors on the external surface of the animal; the proprioceptors within the animal’s musculature are not involved. Such knowledge can contribute to the ecological understanding and to improve management in the *M. rosenbergii* hatchery.

## MATERIALS AND METHODS

### Animal for Experiment

*M. rosenbergii* zoeae were reared to postlarvae PL5 with a commercial diet (CP Aquaculture Limited, Thailand) at water pH 7.4–8.9; salinity 8–12 ppt; and temperature 25.7–27.4ºC at the Shrimp Hatchery of the Borneo Marine Research Institute, Universiti Malaysia Sabah. The postlarvae were gradually acclimated to fresh water (salinity 0) over 7 days in the hatchery before the pH experiment was conducted in wet laboratory. All experimental animals were cared and handled following the guidelines by the World Health Organization (WHO, Geneva, Switzerland); the Malaysian Code of Practice For The Care And Use of Animals For Scientific Purposes; and the Committee for the Update of the Guide for the Care and Use of Laboratory Animals, Institute of Laboratory Animal Research (Committee for the Update of the Guide for the Care and Use of Laboratory Animals, Institute to Laboratory Animal Research 2011).

### pH Treatments

In total, four glass aquaria (60 cm long × 30 cm wide × 30 cm high) were randomly arranged and each of them was filled with deionised tap water to a depth of 25 cm. In every 2 d, the aquaria were randomly rearranged (by means of the table of random numbers) in order to expose the postlarvae in the aquaria to all possible ambient conditions, and to avoid nuisance factors. Each aquarium was introduced with a cubic three-layer polyethylene green netting (34 cm × 24 cm, 12 cm high, 7 mm mesh size) and an air-lift water filtration unit. One of the aquaria was maintained at the ambient water pH 7.5 (control), while the others were adjusted to pH 6, pH 5, and pH 4 by adding in 11.5 mM hydrochloric acid (HCl) so that pH 7.5 was reduced at the rate of 1 pH unit d^−1^. For the pH 4 aquarium, the adjustment took 7 d because after reaching pH 5, HCl was added even more slower in order to reduce pH at the rate of 0.2 pH unit d^−1^ until pH 4. Water in the aquaria was changed twice daily (10% of the volume in morning and 90% in afternoon) with new water of the respective pH (pH-adjusted before addition). Water pH, temperature, salinity, and dissolved oxygen were measured with a pH/ORD/EC/DO tester (Hanna Instruments, HI 9828) at about 30 min after the water replacement. Water temperature ranged 25.4–29.0ºC, dissolved oxygen 5.4–7.0 ppm, and salinity 0.06–0.14 ppt in all aquaria. Each pH aquarium with netting was stocked with 250 postlarvae (initial stage PL5; 5 d post-metamorphosis). The postlarvae were fed the same pelleted diet throughout.

### Shelter Occupancy

Shrimp and prawn postlarvae are positively thigmotactic (Kingsford *et al*. 2002) and are known to cling to twigs, leaf litter, and artificial shelters used as passive fry collecting gear (Kawamura & Bagarinao 1980). Thigmotactic behaviour is driven by the tactile sense. The postlarvae of *M. rosenbergii* crawl at the bottom or cling to submerged objects. In the hatchery, such submerged objects, called shelters, are used to increase the surface area and maximise the stocking density. Occupancy of shelter is a normal behaviour mediated by vision and tactile sense. In our preliminary experiment, postlarvae with normal vision swam or crawled straight to the shelter and occupied it. Blinded postlarvae (whose eyes were painted with white nail polish) swam in random directions after release into the aquaria and collided with the walls and clung to the shelter only after random contact.

Starting at 1 d after the acclimation to each pH treatment, shelter occupancy by *M. rosenbergii* postlarvae in the pH treatments was determined by counting every day at 14:00–15:00 h the number of postlarvae clinging to or resting on the netting. Such counts were made daily for 7 d (i.e., 7 trials). Shelter occupancy was given as the ratio of the number of postlarvae on the shelter to the total number of postlarvae in the aquarium, the latter of which decreased due to mortality over time and by pH treatments. In spite of high mortality in pH 4, the remaining larvae showed no reduction in feeding activity.

### Jumping Response

Another experiment was conducted in which the tactile sense was reckoned as detection of mechanical stimuli by surface epidermal receptor systems. Postlarvae that had been acclimated to different pH for 7 d were assessed for the jumping response. The tests were done in a 500 ml round glass bowl (22.5 cm diameter, 10 cm high) filled 2 cm deep with water of a given pH treatment. The test procedure and the behaviour of postlarvae were recorded with a shadow photography unit that consisted of an overhead projector with a Fresnel lens (APOLLO 8205, ACCO Brands Corporation, USA), a white screen, and a video camera (Olympus digital camera, Tokyo, Japan). The Fresnel lens was covered with a grey plastic sheet (50% transmittance) to reduce the light intensity and inactivate the postlarvae and the test bowl was placed on the plastic sheet. In each test, three random postlarvae were moved from the treatment aquarium into the test bowl with the same water pH. After 20 min rest, the antennae, antennules, or abdomen were tapped with a glass micropipette (heat-pulled glass capillary with a 3 μm tip) at random time intervals >2 s. This procedure was repeated for 3 groups, 35 taps for the first and second groups and 30 taps for the third group, a total 100 taps for each treatment. When a postlarva jumped after a tap, a score of 1 was given and when the postlarva did not jump, a 0 was recorded. Video recording was done for 3–5 min for each group, and the videos were later played back for analysis.

### Statistical Analysis

Since binary data was collected from the shelter occupancy experiment, the binomial theorem was applied to the statistical analysis of shelter occupancy ratio, and the binomial 95% confidence intervals were calculated (Clopper & Pearson 1934; Soper 2014). The Cochran *Q* test (Siegel & Castellan 1988) was applied to the jumping response data which were dichotomized ordinal observations.

## RESULTS AND DISCUSSION

The shelter occupancy ratio was significantly lower at pH 5 and pH 4 than in the control and pH 6 (*P* < 0.05) (Table 1). The postlarvae that occupied the shelter were stationary, clinging to the netting, clearly thigmotactic, whereas those out of the shelter crawled around on the bottom and often swam up to the water surface.

**Figure 1:**
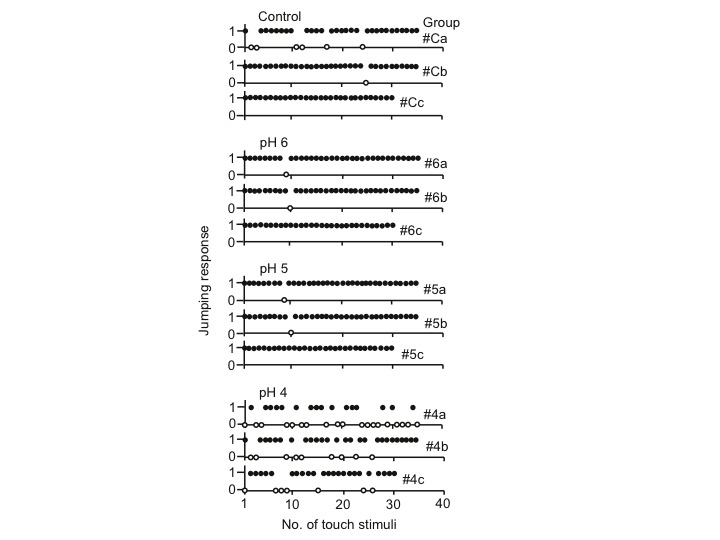
The effect of water pH on the jumping response of *Macrobrachium rosenbergii* postlarvae (3 per group) tapped by a glass micropipette on the antennae, antennules, and abdomen. When a postlarva jumped upon contact, the score given was 1 (solid circles); if not, the score was 0 (open circles)

The sequence of the jumping response of the postlarvae in each pH treatment is shown in Fig. 1. In the control and pH 6 and pH 5 treatments, the jumping response almost always followed a tap, 93–98% of the time (only 2–7% no response), with no evident habituation. However, in the pH 4 treatment, the jumping response of the postlarvae was irregular and of much lower occurrence (65%). The frequency of the jumping response was significantly lower in the pH 4 treatment (Cochran *Q* test, *Q* = 71.714, *P* < 0.001) but not significantly different among control, pH 6 and pH 5 (*Q* = 5.545, 0.10 < *P* < 0.20).

Sudden lowering of the pH causes shock and affects survival and growth of shrimps (Almut & Bamber 2013; Lemonnier *et al*. 2004; Tucker & D’Abrano 2008). In the present study, *M. rosenbergii* postlarvae were gradually acclimated to lower pH and found to have poorer behavioural response to tactile stimuli at pH 4. Arthropod cuticle, including setae, is largely composed of chitin (Wainwright *et al.* 1976), an acid-sensitive material soluble in dilute acids and degraded by several pathways leading to physical property modifications (Percot *et al.* 2003). The low pH water probably physically affects the structure of the setae and inhibits their function. Kawamura *et al*. (2015) reported a thinner carapace in *M. rosenbergii* postlarvae and early juveniles exposed to pH 4. Such decalcification probably impairs mechanoreception. A lower cuticle thickness and reduced cuticle calcium concentration at low pH have been observed in the crayfish *Orconectes virilis* and *Austropotamobius pallipes* (Malley 1980; France 1987; Reese 1963). Among the hermit crabs *Pagurus bernhardus,* a significantly higher ratio (45.7%) failed to exchange shells after exposure to reduced pH, compared to 10.7% at ambient pH (de la Haye *et al*. 2011). The chelar setae of the hermit crab *Pagurus hirsutiusculus* function as mechano- and chemoreceptors sensitive to calcium ions; contact enables it to select an optimal shell size and calcium reception allows it to distinguish shells from pebbles and other objects (Reese 1963; Mesce 1993).

Air pollutions are emitted into the atmosphere from anthropogenic sources and travel across national boundaries. Water is a solvent and natural waters are never pure. Normal pure rainwater usually has a pH of about 6.0 or above (Tucker & D’Abramo 2008). Extreme acidic water in rivers and lakes has been reported: pH 3.2–6.3 in Tasik Chini’s Feeder River in Pahang (Gasim *et al*. 2006); pH 3.2–6.7 at 17 sampling sites in Langat River flowing through oil palm and rubber plantations in Selangor (Juahir *et al*. 2009); pH 3.8–5.4 at Nilai Industrial Park, Negeri Sembilan (Norela *et al*. 2009). Exposure of *M. rosenbergii* postlarvae to lower than pH 5 increases vulnerability to predation. An impaired tactile sense weakens escape jumping in response to touch stimuli conveyed by predators. Inability to cling to hiding or resting places leads to wandering and increased exposure to predators. Bottom substrates provide refuge to crustaceans and mitigate predation (Main 1987; Alberstadt *et al*. 1995). The shelter-seeking behaviour is driven by thigmotaxis (Alberstadt *et al*. 1995). In unstructured shelter the predation rates can be very high, more than 80% among postlarvae and early juveniles of the shore crab *Carcinus maenas* (Moksness *et al*. 1998). Thus, low pH must be avoided in aquaculture systems for *M. rosenbergii* and must be mitigated in natural freshwater systems to prevent adverse effects on wild prawns and other species.

## CONCLUSION

The effect of low pH on the tactile sense of *Macrobrachium rosenbergii* postlarvae was determined in the laboratory by means of two behavioural assays: shelter occupancy and jumping response to touch stimuli by a micropipette. The postlarvae were acclimated to pH 4, pH 5, pH 6 and pH 7.5 (control) in 45 L aquaria before the experiments. Shelter occupancy decreased with pH and was significantly lower at pH 4 and pH 5 than at pH 6 and in the control. The jumping response instantly followed a tap 93–98% of the time in the control and the pH 6 and pH 5 treatments. However, the postlarvae showed significantly lower jumping response (65%) at pH 4, indicating an impaired tactile sense. Low pH 4–5 probably degrades the chitin of the sensory setae and inhibits the surface mechanoreceptors of the prawn postlarvae.

## Figures and Tables

**Table 1:** Shelter occupancy by postlarvae of *Macrobrachium rosenbergii* in seven daily trials after 1 d acclimation to each pH. Each aquarium was initially stocked with 250 postlarvae.

Treatment	Number of postlarvae in the aquria during daily trials							Number of postlarvae occupying the netting shelter during daily trials							Median shelter occupancy ratio (%)*	Binomial 95% confidence interval

	Trial							Trial								

	1	2	3	4	5	6	7	1	2	3	4	5	6	7		
Control	250	250	249	249	249	249	249	193	135	136	78	90	78	87	36.1a	30.2–42.5
pH 6	249	249	249	249	249	249	249	74	105	99	99	111	108	109	42.2a	36.2–48.4
pH 5	243	243	243	243	243	242	242	56	62	71	58	79	53	46	23.9b	18.7–29.7
pH 4	235	202	201	198	184	137	130	38	41	47	44	39	16	14	20.3b	15.0–26.5

* Different letters indicate significant differences at α = 0.05
